# Editorial: Bio-inspired soft locomotion

**DOI:** 10.3389/frobt.2023.1291839

**Published:** 2023-10-02

**Authors:** Onur Ozcan, Murat Reis, Surya G. Nurzaman

**Affiliations:** ^1^ Mechanical Engineering Department, Bilkent University, Ankara, Türkiye; ^2^ Bursa Uludağ University, Bursa, Bursa, Türkiye; ^3^ School of Engineering, Monash University Malaysia, Subang Jaya, Malaysia

**Keywords:** bio-inspiration, bio-inspired, robotic locomotion, locomotion, soft robot

While most conventional mobile robots are made of entirely rigid parts or structures, completely rigid structures are almost non-existent in biological entities capable of locomotion. Studies on soft robots or hybrid soft/hard robots can generate an answer to this dilemma. By using soft materials, mechanisms, and fabrication methods, it is possible to investigate bio-inspired robots that exploits compliance to make better-moving robots. Indeed, biological entities often utilize their body or joint compliances to mechanically adapt to their environments during locomotion or to locomote in a more efficient, faster, or more agile manner. Relevantly, the goal of this Research Topic is to highlight robots that exploit compliant body or joint compliance for a better locomotion performance.

In the first article, Matsumoto et al. propose a design that imitates the spine structure of animals for quadrupedal robots to increase the robots’ locomotion abilities. The experimental results of this design show that the robot with this spine structure can run longer distances and move faster. Forward kinematics calculations revealed that the proposed spine structure could achieve a 1.5 times greater horizontal foot range of motion than a single-joint spine structure. In experiments on a robot equipped with each spine structure, the robot with the proposed spine structure achieved a 1.4 times greater horizontal foot range of motion and 1.9 times greater speed than the one equipped with a single-joint spine structure. In this way, it has been demonstrated that the spine design has the potential to increase the mobility of robots.

A slip-turning motion of musculoskeletal robots is examined by Nipatphonsakun et al. in the second article. Using this rotation technique and the active control of the stiffness of the joints, it is aimed that the robots gain more rotational angles and increase their mobility and stability. The bipedal robot realized the slip-turning motion on toe joints about its yaw axis, while all eight joints were placed on the pitch axis. The foot with toe joint has enabled the heel-off motion of the musculoskeletal robot and could reduce the foot contact area during the turning motion compared to the rigid foot with a fixed toe, resulting in a reduction of frictional torque, minimizing its power, and an increase in the rotational angle. The second experiment in the study proved that by using the PIM to restrain the toe joint, the robot could prevent the over-dorsiflexion of the toe, which can contribute to the improvement of static postural stability in the anterior-posterior direction. Meanwhile, the active toe could generate even stronger propulsion, which can be useful in a quick motion. The results indicate that the inner muscles of the knuckles and soles of the feet contribute to the gliding motion.

In the third article, Aboufazeli et al. introduce an online learning algorithm inspired by the adaptive behaviours of animals, focusing on legged robots. It aims to enhance energy efficiency and stability in legged robot locomotion through real-time leg stiffness and stride angle adjustments, akin to how animals optimize their gait parameters in response to changing terrain. The algorithm utilizes an approximate stochastic gradient method for dynamic parameter changes and offers several benefits: computational efficiency, no need for pre-training, model-free adaptability, and applicability to various robots and gaits. The algorithm’s effectiveness is demonstrated through simulations and experiments on a quadruped robot platform equipped with compliant legs. The robot’s leg stiffness is controlled by varying pneumatic pressure in each leg, while the stride angle is adjusted to optimize energy consumption. Compared to traditional optimization approaches, this algorithm operates in real-time, adapting parameters to ensure efficient locomotion even in varying conditions.

The fourth article, by Buckley et al., focuses on the significant role of tails in quadrupedal locomotion, both in biological organisms and bio-inspired legged robots. While tails in animals serve various functions such as propulsion, balance, and stability during movements like walking, running, and climbing, they are often overlooked in the field of legged robotics due to design and control challenges. The authors investigate the impact of a variable stiffness robotic tail on the performance of a sprawling quadruped robot, aiming to enhance stability and maneuverability across different environments. They integrate a flexible cable-driven tail with a servo motor into the robot’s design and demonstrate that tail-controlled stiffness improves locomotion stability on rough terrain and climbing ability compared to no tail or a rigid tail. The findings underscore the importance of constant ground support offered by the flexible tail, resulting in enhanced gait predictability, increased speed, and better efficiency. The flexible tail also allows the robot to adapt across various terrains, indicating its potential to address challenges in quadrupedal navigation in complex environments.

In the fifth article, Ishiguro et al. delves into the significant role of wing veins in micro-flapping aerial vehicles inspired by insects like bees and flies. These insects possess flexible wings with veins that enhance flight efficiency and resilience. The study explores the integration of wing veins into soft wings for micro-flapping aerial vehicles, aiming to optimize flight performance. The researchers design and evaluate prototypes of soft wings with varying wing areas and vein patterns in both wing-chord and wing-span directions. The results highlight that the force generated during wing flapping is not solely determined by wing area; instead, it's influenced by the wing vein pattern. Wings with wing-chord veins generate more force compared to those with wing-span veins. However, when considering a specific wing area, wings with crossed wing veins, incorporating both wing-span and wing-chord veins, produce higher force. The study emphasizes the complex interplay between wing area and vein orientation and underscores the potential for optimized wing designs to enhance the performance of micro-flapping aerial vehicles.

Finally, in the last article, Godon et al. review solutions for the locomotion on frictionally yielding media—non-Newtonian fluids that significantly deform under stress and do not recover their original shape, as seen in mud, snow, soil, and sand. While some robots have been designed for such substrates, there is a lack of prototypes developed outside research labs. This paper comprehensively examines biology and robotics literature to discern principles facilitating movement on yielding terrains. The analysis categorizes animal and robot locomotion based on mechanical principles and the nature of contact. These categories include discrete contact, continuous contact above the material, and contact through the medium. The research unveils diverse hardware solutions and motion strategies that enable various robots and animals to navigate yielding environments. The paper also underscores that a higher level of abstraction aids in transferring solutions to the robotics domain, even when the robot is not explicitly bio-inspired. The primary contribution is an insightful review of biology and robotics literature, identifying locomotion principles for potential application in designing robots for yielding environments and a compendium of existing solutions that can guide locomotion on such grounds.

